# Asymptomatic HyperCKemia: A Diagnostic Trap

**DOI:** 10.7759/cureus.12791

**Published:** 2021-01-19

**Authors:** Safia Shaikh, Alan Gonzalez- Zapata, Abdul Mujeeb, Abdul Rauf, Dany Thekkemuriyil,

**Affiliations:** 1 Internal Medicine, SSM Health St Mary's Hospital, St Louis, USA; 2 Internal Medicine, SSM Health St. Mary's Hospital, St. Louis, USA; 3 Urology, Jinnah Postgraduate Medical Centre, Karachi, PAK; 4 Internal Medicine- Rheumatology, SSM Health St. Mary's Hospital, St. Louis, USA

**Keywords:** macroenzyme, creatine kinase, hyperckemia, macrockemia, ck electrophoresis

## Abstract

Elevated creatine kinase (CK) levels are the most sensitive indicator of muscle injury. Levels >5000 U/L warns physicians to initiate aggressive hydration and prevent renal failure. We present a rare case of asymptomatic hyperCkemia with levels >80 times the upper limit of normal (ULN), refractory to fluid resuscitation. Our patient was found to have elevated macroenzymes- macroCkemia, causing decreased clearance of CK. The objective of this case report is to bring to attention a rare and benign cause of CK elevation which can lead to diagnostic and therapeutic errors.

## Introduction

HyperCKemia is defined as persistent CK levels > 210 U/L in women, ≥400 U/L in men <50 years and ≥280 U/L in men ≥50 years (reference values according to the Nordic Reference Interval Project) [1]. CK is a dimer molecule and occurs in three distinct isoenzyme forms electrophoretically (MM, BB, and MB). In skeletal muscles, MM is the predominant fraction with 99%, while MB constitutes a small fraction. Common causes of elevated CK include trauma, exercise, inflammatory disorders, myopathies, medications (commonly statins). It is also seen as secondary muscle involvement, in neurogenic disorders such as amyotrophic lateral sclerosis, hereditary spinal muscular atrophies. Elevated CK level is of toxicological significance and is widely used to detect muscle damage.

## Case presentation

**Figure 1:**
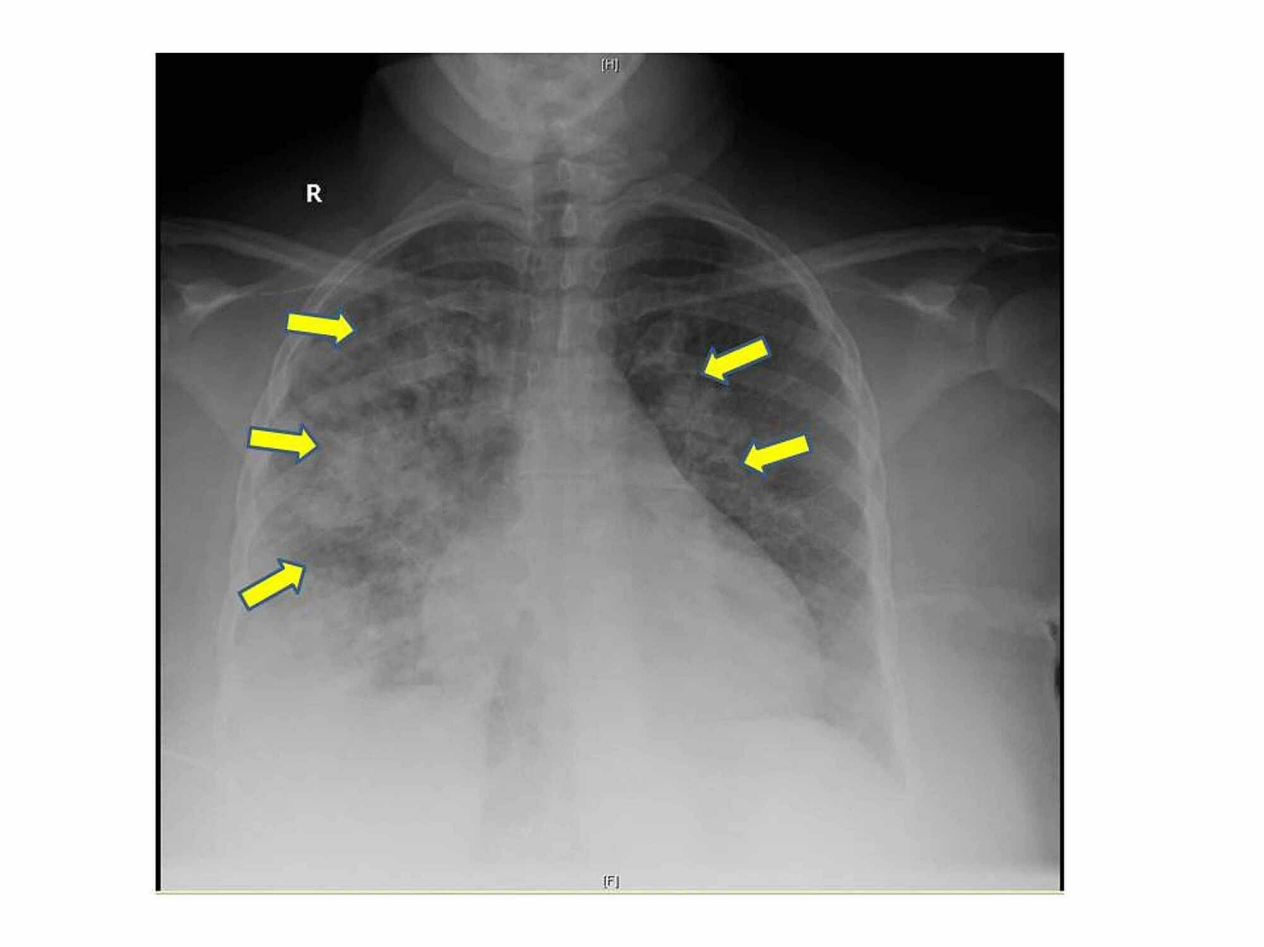
Chest X-ray of the patient showing diffuse bilateral infiltrates.

**Figure 2:**
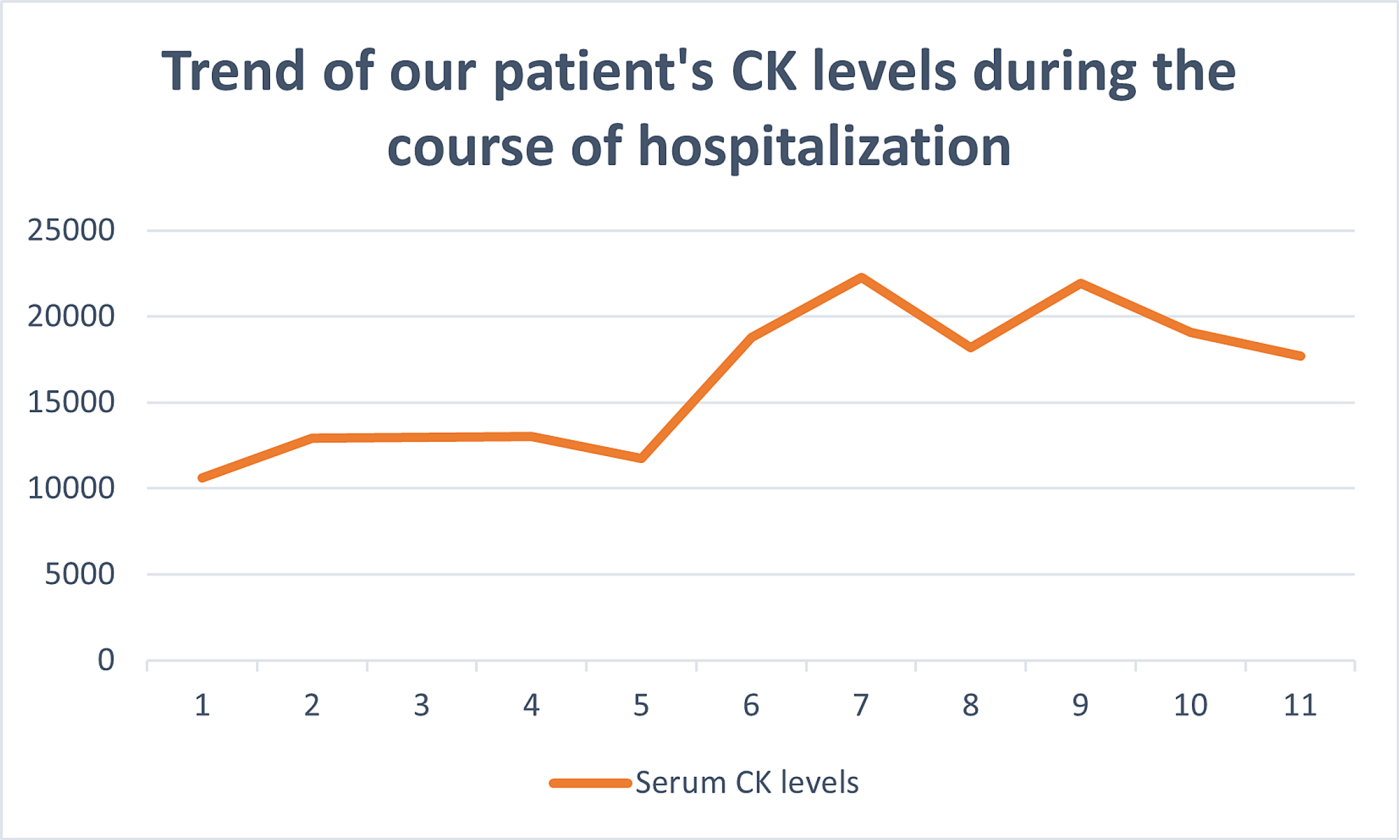
Trend of CK enzyme in our patient during the course of hospitalization.

A 43-year-old female was admitted with acute hypoxic respiratory failure, requiring 15 litres per minute of oxygen. Physical exam was significant for wheezing in lungs bilaterally. Electrocardiogram showed normal sinus rhythm. Troponin and electrolytes were in normal range. She had normocytic anemia with hemoglobin 10.8 g/dl (normal range 12.0-15.6 g/dl), leukocytes 7.8 X 10^9^/L( normal range 4.4- 10.7 10^9^/L) , serum procalcitonin at 0.10 ng/mL (normal range < 0.10 ng/mL). Arterial blood gases showed respiratory acidosis. Chest X-ray showed extensive bilateral lung infiltrates (shown in figure-1). Workup for infectious causes was negative. She was managed empirically for community acquired pneumonia with broad spectrum antibiotics. On day 3 of hospital stay, her symptoms and oxygen requirement improved. However, she was noted to have hyperkalemia (potassium: 5.8 mmol/L) and elevated CK at 10,000 U/L ( normal range 24-173 U/L). She denied any history of myalgias, muscle weakness, trauma, recent exercise, illicit drug use, and statins. An impression of rhabdomyolysis was made and aggressively hydrated. Surprisingly, the CK level increased despite resuscitation (Figure 2), raising diagnostic curiosity. Detailed inflammatory workup including aldolase, myositis antibody panel and anti-neutrophil cytoplasmic antibodies was negative. Her course of hospitalization was complicated by volume overload requiring aggressive diuresis. A detailed discussion with the patient was held explaining enzyme abnormality and risk versus benefits of invasive workup. She refused further investigations including biopsy and imaging. A CK electrophoresis was ordered and the patient was discharged to follow up as an outpatient. Surprisingly, her isoenzyme panel showed CK MM with type 1 macroCK-macroCKemia (Table 1). CK levels repeated after 10 days showed near normal levels. On post-hopital follow up, subsequent normalization of enzyme levels were seen.

**Table 1:** CK iso-enzyme panel of our patient

Component (Reference range and units)	Results
Serum CK levels (24 - 173 U/L)	22552 U/L
CK-MM% (97 - 100 %)	99
Macro type 1 (Not Observed %)	1
Macro type 2 (Not Observed %)	0
CK-MB (0 - 3 %)	0
CK-BB (0%)	0

## Discussion

Macroenzymes are the serum enzymes that have a higher molecular mass (>200KDa) than the corresponding enzymes normally found in serum (80-KDa) under physiological or pathological conditions [2,3]. They are more slowly cleared from the circulation than the normal enzyme CK, leading to persistently elevated levels. When quantitative total enzyme assays are used, macroenzymes are indistinguishable from the normal enzymes, causing an elevation of the total assay.

Macro CK is the most common macro enzyme. Others are macro amylase, macro lactate dehydrogenase (Macro-LDH), macro-alkaline phosphatase (Macro-ALP) and macro-aspartate transaminase (Macro-AST). Usually, serum electrophoresis can identify macroenzymes, but if inconclusive, either gel electrophoresis or an immunologic assay should be used [3].

In serum, macro CK occurred in two major forms: macro CK type 1, a complex composed of an immunoglobulin (commonly IgG or IgA), and macro type 2, an oligomeric mitochondrial creatine kinase released due to tissue necrosis [4].

Certain conditions favor the presence of macroenzymes: 1. absence of symptoms, 2. symptoms atypical for abnormal enzyme levels, 3. an isolated and persistently increased serum enzyme level [2,3]. Failure to suspect the macroenzyme early in diagnostic workup can lead to undue, expensive, and invasive tests in search of alternate diagnosis [3].

No evidence convincingly indicates that macro enzymes have prognostic value for disease or impending death [5]. Nonetheless, macro type 1 and type 2 have been reported to be associated with autoimmune diseases or malignant lesions of stomach, breast, and prostate [3]. Macro CK has been proposed to be a tumor marker for colorectal cancer [6]. Also, macro CK and LDH have been found to occur in presence of acute or remote MI. The alteration of self antigen theory is a plausible explanation for this association. It states that damaged tissues release enzymes that can be altered by inflammatory hydrolytic proteases making them immunogenic. The explanation also fits our patient’s finding of elevated total assay in the hospital, which was normalized at home, after inflammation subsided.

It is also reported that macro-enzymes can persist and even recur after initial resolution. Hsiao et al. described the recurrence of macro CK enzyme elevation after one month in patients with myopathy. It is, therefore, of paramount importance to document in the patient’s chart to avoid repeated workup [7]. The importance of early diagnosis and documentation cannot be emphasized enough. Wediner and colleagues described an elderly lady who underwent multiple hospitalizations, repeated liver biopsies, gastroenterologists’ consultations for isolated increased AST, ultimately attributed to Macro-AST [8].

## Conclusions

Macro CK is a rare but important cause of CK elevation. Macroenzymes are indistinguishable from normal enzyme levels on a regular quantitative assay. It could lead to a false diagnosis of rhabdomyolysis (CK-MM), myocardial infarction (CK-MB) or pancreatitis (amylase). Macroenzymes should always be suspected in patients with isolated laboratory enzyme elevation without typical symptoms. Once identified, the data should be entered into the patient's chart and communicated to the providers to avoid unnecessary invasive repeated diagnostic and therapeutic interventions.

## References

[1] H. Lilleng, K. Abeler, SH Johnsen (2011). Variation of serum creatine kinase (CK) levels and prevalence of persistent hyperCKemia in a Norwegian normal population. The Tromsø study. Neuromuscul Disord.

[2] Galarraga B, Sinclair D, Fahie‐Wilson MN, McCrae FC, Hull RG, Ledingham JM (2003). A rare but important cause for a raised serum creatine kinase concentration: two case reports and a literature review. Rheumatology.

[3] Galasso PJ, Litin SC, O'Brien JF (1993). The macroenzymes: a clinical review. Mayo Clin Proc.

[4] Mifflin TE, Bruns DE, Wrotnoski U, MacMillan RH, Stallings RG, Felder RA, Herold DA (1985). University of Virginia case conference. Macroamylase, macro creatine kinase, and other macroenzymes. Clin Chem.

[5] Lee KN, Csako G, Bernhardt P, Elin RJ (1994). Relevance of macro creatine kinase type 1 and type 2 isoenzymes to laboratory and clinical data. Clin Chem.

[6] Mercer DW, Talamo TS (1985). Multiple markers of malignancy in sera of patients with colorectal carcinoma: preliminary clinical studies. Clin Chem.

[7] Hsiao JF, Ning HC, Gu PW, Lin WY, Chu PH (2008). Clinical role of recurrently elevated macro creatine kinase type 1. J Clin Lab Anal.

[8] Weidner N, Lott JA, Yale VD, Wahl RL, Little RA (1983). Immunoglobulin-complexed aspartate aminotransferase. Clin Chem.

